# Point-of-care ultrasound assessment of the inferior vena cava distensibility index in mechanically ventilated children in the operating room

**DOI:** 10.3906/sag-2006-300

**Published:** 2021-06-28

**Authors:** Dinçer YILDIZDAŞ, Özden ÖZGÜR HOROZ, Ahmet YÖNTEM, Faruk EKİNCİ, Nagehan ASLAN, Demet LAFLI TUNAY, Murat Türkeün ILGINEL

**Affiliations:** 1 Department of Pediatric Intensive Care Unit, Medical Faculty, Çukurova University, Adana Turkey; 2 Department of Anesthesiology and Reanimation, Medical Faculty, Çukurova University, Adana Turkey

**Keywords:** Distensibility index, mechanically ventilation, children

## Abstract

**Background and aim:**

Point-of-care ultrasound imaging of the inferior vena cava distensibility index is a potential indicator for determining fluid overload and dehydration in the mechanically ventilated patients. Data on inferior vena cava distensibility index and inferior vena cava distensibility variability are limited in mechanically ventilated pediatric patients. That is why our aim in this study was to measure inferior vena cava distensibility index and to obtain mean values in pediatric patients, ventilated in the operating room before the ambulatory surgical procedure started.

**Materials and methods:**

This crosssectional study was performed between February 2019 and February 2020. Ultrasonographic measurements were performed in a total of 125 children.

**Results:**

In a period of 13 months, the measurements were performed in a total of 125 children, of which 120 (62.5% male) met the criteria and were included in the study. Overall inferior vena cava distensibility index (%): mean   SD: 6.8   4.0, median (min–max): 5.7 (1.4–19.6), IQR: 3.8–8.7. Overall inferior vena cava distensibility variability (%): mean   SD: 6.5   3.7, median (min–max): 5.5 (1.4–17.8), IQR: 3.7–8.4.

**Conclusion:**

Our study is the largest series of children in the literature in which inferior vena cava distensibility index measurements were investigated.

## 1. Introduction 

Fluid management is a critical issue in intensive care units and operating rooms. In order to evaluate the volume status of the patients, patient history, vital signs, physical examination, laboratory results, and other more invasive methods have been used [1]. The American College of Critical Care Medicine stressed early and aggressive fluid resuscitation in the guideline on hemodynamic support of pediatric and neonatal shock in 2002 [2]. However, it is beneficial to be careful in terms of excessive fluid resuscitation in children, undergoing volume replacement, because various complications, including intraabdominal hypertension, may occur in patients who receive excessive fluids [3]. For these reasons, clinicians are trying to find the best tool to properly perform intravenous fluid replacement. Indices such as central venous pressure have been shown to lack a good evidence for accuracy in determining intravascular volume sensitivity [4]. In recent years, the use of ultrasonography has become increasingly widespread in intensive care units. In this way, a noninvasive, painless, cheap, easy, and objective method, specifically in adult patients, could be performed. One of the benefits of ultrasonography is to evaluate the volume status of patients with the help of inferior vena cava (IVC) (that receives all the blood from below the diaphragm) diameter and vena cava collapsibility index (the percentage decrease in IVC diameter with inspiration) measurements in the nonventilated spontaneously breathing patients [5]. However, opposite physiology occurs during positive pressure ventilation. Therefore, the distensibility index of the IVC (IVC-DI) is used in patients with mechanical ventilation [6]. IVC-DI = [(maximum diameter–minimum diameter)/(minimum diameter)] × 100) and the IVC distensibility variability (IVC-DV) = [(maximum diameter – minimum diameter)/(mean diameter)] × 100) in mechanically ventilated adult patients may predict fluid responsiveness [7,8]. 

Data on IVC-DI and IVC-DV are limited in pediatric patients with mechanical ventilation. Basu et al. found that IVC-DI and IVC-DV significantly correlated with fluid overload in 50 children on mechanical ventilation [9]. Achar et al. conducted a prospective study with 42 children on mechanical ventilation undergoing general anesthesia for elective surgery and they reported that IVC-DI and aortic flow peak velocity index are reliable indices of fluid responsiveness in children [10]. That is why our aim in this study was to measure IVC-DI and IVC-DV, and to determine the reference values in pediatric patients ventilated in the operating room before the ambulatory surgical procedure.

## 2. Material and methods

This cross-sectional study was performed between February 2019 to February 2020. The study was carried out at the Çukurova University operating room. Ultrasonographic measurements were performed in a total of 125 children ages ranging from 1 month to 18 years of age. Patients with a clinical history and objective findings of hypovolemia, vomiting, diarrhea, fever, abdominal pain, malnutrition, chronic diseases including renal insufficiency, diabetes, cardiac disease, liver disease, chronic obstructive lung disease, and children whose weight was below 60% according to their age were excluded from the study. Participants in whom the IVC could not be well visualized were also excluded since essential outcome measurements could not be obtained. A total of 120 children who met the criteria were included in the study. 

Approval for the study was obtained from Çukurova University Faculty of Medicine Clinical Research Ethics Committee (date: 04/01/2019, number: 84/6). Written consent (from their guardians) was obtained for all children. 

Data collection was performed by two investigators (A.Y. and N.A.), who were coordinated by a pediatric intensive care specialist, and who also underwent an 8 h training course consisting both of theoretical and practical experience and completed more than 300 supervised scans in a variety of pediatric intensive care unit applications before the initiation of the study. These researchers were also approved by the faculty member of the radiology department. Before the enrollment of the overall population, a sample of 30 subjects were evaluated by raters in order to assess the intra- and inter operator reliability; Lin’s concordance coefficient resulted to be excellent, with values over 0.90. 

Clinical and demographic characteristics of participants such as age (months), body weight (kg), height (cm), body mass index (BMI) (kg/m²), and body surface area (BSA) (m2) (Haycock formula) were recorded. 

Sonographic measurements of IVC were performed 5 min after intubation with an endotracheal tube suitable for their age. All sonographic measurements were done in the operating room before the surgery started. During ultrasonography, all patients were mechanically ventilated in the supine position, using volume-controlled ventilation. An inspiratory-expiratory ratio of 1:2, a respiratory rate for age, a tidal volume of 7–8 mL/kg, and a positive end-expiratory pressure (PEEP) of 5 cm H2O were applied.

Ultrasound examinations of the IVC were performed with Mindray ultrasound system (Mindray, Shenzhen, China), using a 2.1–5.1 MHz phased array transducer. Measurements were taken over 3 to 4 cycles during positive pressure ventilation. A transducer was placed just below the level of xiphoid bone. To obtain the sagittal image, the probe was placed in the subxiphoid area and the liver was used as an acoustic window. The IVC entry into the atrium was identified. In the M-mode, maximum IVC diameter on inspiration and minimum IVC diameter on expiration (Figure) was recorded just after the point where the hepatic veins were poured into the IVC. Vena cava distensibility index and distensibility variability were calculated with the following formulas: IVC-DI = (max diameter–min diameter)/(min diameter) × 100, IVC-DV = [(maximum diameter – minimum diameter)/(mean diameter)] × 100).

**Figure:**
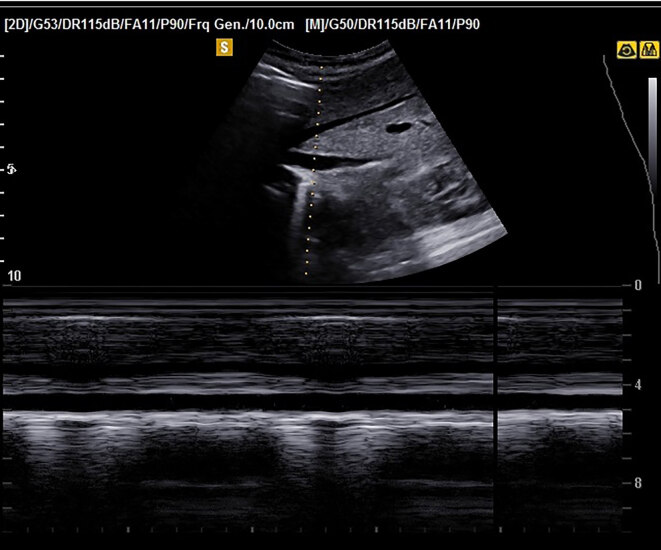
Sagittal view of the IVC in the subxiphoid region.

The children were divided into 4 groups, according to their age, as group 1: 0–24 months (n = 20), group 2: 25–48 months (n = 19), group 3: 49–120 months (n = 44), group 4: ≥ 121 months (n = 37).

### 2.1. Statistical analysis

All analyses were performed using the Statistics Version 20.0 statistical software package (SPSS, IBM Corp, Armonk, NY, USA). Categorical variables were expressed as numbers and percentages, whereas continuous variables were summarized as mean and standard deviation and as median and minimum-maximum values where appropriate. The normality of distribution for continuous variables was confirmed with the Kolmogorov–Smirnov test. The normality of distribution for continuous variables was confirmed with the Shapiro–Wilk test. To evaluate the correlations between measurements, the Pearson correlation coefficient was used. The statistical level of significance for all tests was considered to be 0.05 .

## 3. Results

In a period of 13 months, measurements were performed in total on 125 children, of which 120 (62.5% male) met the criteria and were included in the study. Five were excluded due to suboptimal visualization of the IVC. 

In the perioperative period, the vital signs of the patients were in the normal range according to their age. Demographic characteristics of the population and descriptive statistics with mean and SD, median, minimum, maximum for all sonographic measurements are listed in Table 1. The majority of the operations were performed by otorhinolaryngology, urology, and pediatric surgery departments. Ambulatory surgeries performed to our patients are shown in Table 2. Overall IVC-DI (%): mean ± SD: 6.8 ± 4.0, median (min-max): 5.7 (1.4–19.6), IQR: 3.8–8.7. Overall IVC-DV (%): mean ± SD: 6.5 ± 3.7, median (min-max): 5.5 (1.4–17.8), IQR: 3.7–8.4. 

**Table 1:** Demographic characteristics of the population and for all sonographic measurements.

	Overall (n = 120) mean SD median (min-max)(25–75p)	Age group 1(n = 20)mean SDmedian (min-max)(25–75p)	Age group 2(n = 19)mean SDmedian (min-max)(25–75p)	Age group 3(n = 44)mean SDmedian (min-max)(25–75p)	Age group 4(n = 37)mean SDmedian (min-max)(25–75p)
Age (months)	88.4 ± 58.1 86.0 (3.0–215.0) 36.0–134.7	16 ± 7.0 17.5 (3.0–24.0) 10–22	35.7 ± 6.1 36 (27–47) 31–40	83.3 ± 21.3 86.0 (49.0–118.0) 61.3–103.3	160.7 ± 28.0 153.0 (121.0–215.0) 137.0–186.0
M/F	75/45	11/9	14/5	30/14	20/17
Weight (kg)	26.8 ± 17.0 21.0 (4.0–95.0) 14.0–37.0	9.6 ± 2.8 10.0 (4.0–13.0) (8.3–11.0)	14.8 ± 2.4 14.0 (11.0–20.0) 13.0–17	23.2 ± 7.8 21.5 (14.0–48.0) 18.0–25.0	45.7 ± 14.7 46.0 (23.0–95.0) 23.0–95.0
Height (cm)	119.0 ± 30.1 118.0 (56.0–183.0) 94.0–-142.0	75.5 ± 9.9 78.5 (56.0–90.0) 70.0–84.8	95.8 ± 6.1 94.0 (88.0–108.0) 91.0–98.0	119.3 ± 12.8 118.5 (83.0–145.0) 108.3–127.8	153.7 ± 14.4 155.0 (130.0–183.0) 143.5–163.5
BMI(kg/m2)	16.9 ± 3.0 16.3 (11.1–28.4) 15.1–18.4	15.9 ± 1.7 16.2 (11.1–18.4) 15.3–16.8	16.1 ± 1.6 16.2 (12.9–20.1) 14.8–16.9	15.9 ± 2.4 15.4 (12.8–22.8) 14.6–16.4	19.3 ± 3.6 18.8 (12.0–28.4) 17.1–21.7
BSA(m2)	0.93 ± 0.4 00.8 (0.25–2.20) 0.60–1.24	0.45 ± 0.1 00.47 (0.25–0.57) 0.41–0.51	0.62 ± 0.0 70.62 (0.50–0.76) 0.58–0.66	0.87 ± 0.1 80.83 (0.57–1.39) 0.72–0.94	1.40 ± 0.2 81.41 (0.91–2.20) 1.20–1.60
Heart rate	108 ± 23 108 (70–165) 90–125	133 ± 13 132 (110–160) 120–143	115 ± 20 110 (75–-145) 105–135	102 ± 16 100 (75–135 )90–110	95 ± 22 90 (70–165) 79–102
Respiratory rate	26 ± 4 25 (15–35) 25–30	31 ± 2 30 (30–35) 30–35	29 ± 2 30 (25–30) 30–30	26 ± 2 25 (25–30) 25–25	21 ± 3 20 (15–25) 20–25
Systolic blood pressure	106 ± 15 109 (75–150) 95–115	97 ± 16 95 (75–125) 80–110	95 ± 8 90 (85–110) 90–100	104 ± 10 105 (80–130) 98–110	119 ±14 120 (90–150) 110–125
Diastolic blood pressure	65 ± 12 65 (40–110) 55–75	57 ± 14 57 (40–85) 45–70	58 ± 7 60 (50–80) 55–60	63 ± 9 65 (50–80) 55–70	76 ± 11 75 (55–110) 70–85
Tidal volume	215 ± 131 175 (10–700) 125–300	85 ± 25 88 (35–125) 75–100	125 ± 23 125 (100–175) 100–125	188 ± 63 175 (110–400) 150–200	368 ± 110 350 (175–700) 300–450
IVC max (cm)	1.18 ± 0.411.16 (0.24–2.13)0.85–1.47	0.60 ± 0.190.63 (0.24–0.92)0.49–0.75	0.96 ± 0.150.98 (0.75–1.30)0.83–-1.06	1.18 ± 0.231.17 (0.71–1.69)1.01–1.33	1.61 ± 0.271.61 (1.13–2.13)1.39–1.85
IVC min (cm)	1.11 ± 0.40 1.10 (0.21–2.00) 0.80–1.41	0.55 ± 0.17 0.59 (0.21–0.84) 0.45–0.67	0.90 ± 0.15 0.92 (0.70–1.25) 0.75–0.97	1.11 ± 0.22 1.11 (0.64–-1.59) 0.95–1.27	1.52 ± 0.26 1.52 (0.96–2.00) 1.32–1.72
IVC-DI (%)	6.8 ± 4.0 5.7 (1.4–19.6) 3.8–8.7	9.4 ± 4.8 7.5 (3.119.6) 5.9–13	6.8 ± 3.7 5.3 (2.0–13.3) 3.8–10.9	6.3 ± 3.6 5.3 (1.4–16.1) 3.6–9.1	5.8 ± 3.6 4.9 (1.4–17.7) 3.3–7.5
IVC-DV(%)	6.5 ± 3.7 5.5 (1.4–17.8) 3.7–8.4	8.9 ± 4.3 7.2 (3.0–17.8) 5.7–12.2	6.5 ± 3.4 5.2 (2.0–12.5) 3.7–10.3	6.1 ± 3.4 5.1 (1.4–14.9) 3.6–8.7	5.6 ± 3.3 4.8 (1.4–16.3) 3.3–7.3

**Table 2:** Types of ambulatory surgery.

Operation	n = 120 n (%)
Otorhinolaryngologycal	37 (30.8)
Cochlear implant	24 (20.0)
Tonsillectomy	6 (5.0)
Thyroid biopsy	3 (2.5)
Adenoidectomy	2 (1.7)
Oral mass excision	2 (1.7)
Urologycal	24 (20.0)
Percutaneous nephrolithotomy	10 (8.3)
Cystoscopy	6 (5.0)
Hypospadias	5 (4.2)
Orchiopexy	3 (2.5)
Pediatric surgery	23 (19.2)
Endoscopy	12 (10.0)
Inguinal hernia	3 (2.5)
Laparoscopy	2 (1.7)
Appendectomy	1 (0.8)
Gonad scan	1 (0.8)
Adrenal mass	1 (0.8)
Cholecystectomy	1 (0.8)
Orchiopexy	1 (0.8)
Rectal biopsy	1 (0.8)
Orthopedics	17 (24.2)
Fracture	9 (7.5)
Limb deformity	4 (3.3)
Mass excision	2 (1.7)
Hip dislocation	2 (1.7)
Ophthalmologycal (strabismus)	9 (7.5)
Cranial	6 (5.0)
Intracranial mass	5 (4.2)
Ventriculoperitoneal shunt	1 (0.8)
Thoracic surgery (thoracotomy)	2 (1.7)
Miscellaneous (stem cell transplant)	2 (1.7)

We found that IVC-max and IVC-min were positively correlated with age, body weight, height, BSA, and BMI (P < 0.001). IVC-DI and IVC-DV were also correlated weakly and negatively with age, body weight, height, and BSA (P < 0.05), but no correlation with BMI was detected (P > 0.05). Correlation coefficients (r) between anthropometric and sonographic measurements were shown in Table 3. 

**Table 3:** TCorrelation coefficients (r) between anthropometric and sonographic measurements.

	IVC max (cm)rP	IVC min (cm)rP	IVC-DI (%)rP	IVC-DV (%)rP
Age (m)	0.847<0.001	0.844<0.001	–0.2070.023	–0.2080.023
Weight (kg)	0.833<0.001	0.833<0.001	–0.2000.028	–0.2000.028
Height (cm)	0.903<0.001	0.899<0.001	–0.2250.014	–0.2250.013
BMI (kg/m2)	0.510<0.001	0.510 <0.001	–0.1140.217	–0.1120.223
BSA (m2)	0.877<0.001	0.875<0.001	–0.2090.022	–0.2090.022

## 4. Discussion

In this study, IVC-DI and IVC-DV measurements were performed on intubated patients who underwent ambulatory surgical procedures. Our aim was to detect the mean IVC-DI and IVC-DV values in these children who underwent mechanical ventilation in operating room. 

We know that, a 10% fluid overload in intensive care patients is associated with mortality. The American College of Critical Care Medicine practice guidelines for pediatric and neonatal septic shock recommend intervention when a patient reaches a 10% volume overload [1]. The percent fluid overload by weight and percent fluid overload by volume are parameters used when determining fluid load and renal replacement therapy in critically ill patients [9]. But, potential limitations include an inability to account for insensible losses in volume-based calculations and safety and inaccuracy issues for weight-based calculations [11]. At this stage, point-of-care ultrasound (POCUS) imaging of the IVC, which is a potential indicator for determining fluid overload, has directed clinicians’ attention to this area. POCUS imaging is a non-invasive, painless, cheap, easy, and objective method. A previous study has shown that IVC-DI and IVC-DV significantly correlated with the percentage fluid overload by weight and may have potential as markers for fluid overload in mechanically ventilated critically ill pediatric patients [9]. 

In two metaanalysis studies evaluating IVC-DI and IVC-DV thresholds in mechanically ventilated adult patients, cut-offs of 18% for the IVC-DI and 12% for the IVC-DV have been used to distinguish fluid responsiveness [12,13]. In our study, overall IVC-DI (%): mean ± SD: 6.8 ± 4.0, median (min-max): 5.7 (1.4–19.6), IQR: 3.8–8.7. Overall IVC-DV (%): mean ± SD: 6.5 ± 3.7, median (min-max): 5.5 (1.4–17.8), IQR: 3.7–8.4. We hope that the mean IVC-DI and IVC-DV values obtained from 120 children who underwent ambulatory surgery and ventilated with 7–8 mL/kg tidal volume and 5 cm H2O PEEP pressure will form the basis for future pediatric studies about the correlation between IVC measurements and fluid status for studies in this field in children are very limited.

During positive pressure ventilation, when the pressure outside the intrathoracic vessels exceeds the pressure inside, the intrathoracic parts of the IVC collapse, and the extrathoracic parts become distend. Then, the distensibility indices of IVC increase. In the inspiratory phase of positive pressure ventilation, pleural and right atrial pressures increase and venous return to the heart decreases. Thus, while the diameter of IVC increases during inspiration, it decreases during expiration. IVC-DI measures respiratory variations of the maximum and minimum IVC diameters. Bilgili et al. reported the cut-off value of response to intravenous fluids was determined to be IVC-DI > 22.73% with a sensitivity of 100% and specificity of 100% [15]. The author explained that, relatively high IVC-DI values in children compared to adults can be explained by children’s high thoracic and lung compliance, so the respiratory effects of increased intrathoracic pressure may only cause slight changes in IVC distensibility [15]. Also, Achar et al. found that a threshold value of IVC-DI 23.5% allowed the distinction between the responder and none responder [10]. We obtained lower values in our study: overall IVC-DI: mean ± SD: 6.8 ± 4.0 and overall IVC-DV (%): mean ± SD: 6.5 ± 3.7. Our measurements were lower than both Achar et al. and Bilgili et al.’s measurements, and this may be because we had patients as young as three months old [10,14]. To our knowledge, it was shown that there is a correlation between age and maximum IVC diameter in earlier studies [15–19].

In ventilated adults with hemodynamically unstable subarachnoid hemorrhage, it was shown that the most appropriate test (between mean arterial pressure, intracranial pressure, cerebral perfusion pressure, cardiac index, central venous pressure, stroke volume variation, and IVC-DI) in the evaluation of the response to the fluid above 16% was IVC-DI, with a sensitivity of 70.5% and a specificity of 100%. In the same study, sensitivity 94%, however with a lower specificity 58.3% and 10.6% cut-off value also has been reported [20]. This cut-off value is considerably lower than the previously reported children’s value [10,14]. Although Bilgili et al. claim that children may have high IVC-DI values due to high thoracic and lung compliance, we determined the IVC-DI value of all participants as 6.8 ± 4.0% in our study. The average IVC-DI values in our pediatric patients, who are on mechanical ventilation, have no fluid loss, and have 5 cm H2O PEEP pressure and 7–8 mL/kg tidal volume, are like this and we think that these data will provide a basis for future studies in which the response to fluid will be evaluated in pediatric patients.

Although data on adult surgical patients are limited, a recent meta-analysis indicated that the IVC-DI threshold value ranged from 12% to 40% in studies. However, the authors concluded that these studies consisted of heterogeneous patient populations, and their results were contradictory [22]. Therefore, in recent studies with homogeneous patient groups in adult patients, the threshold values were reported to be between 12% and 18% [8,20,22,23]. In a recently published review, it has been reported that “ın ventilated patients without spontaneous respiratory efforts, the mean IVC-DI threshold was 15% (range, 12%–21%)” [12]. However, it is a fact that many studies are required to give IVC-DI threshold values in children. 

Assessing and monitoring the fluid status of perioperative pediatric patients to maintain hemodynamic stability is of great importance for the surgeons as well as the anesthesiologist. For this purpose, vital signs should be monitored closely. Sometimes fasting times can take longer than expected for the planned surgery. Prolonged fasting time causes perioperative complications in especially young children who are more prone to dehydration. In our study, the patients were operated at the scheduled time and there was no significant delay. Also, during the procedure, both pulse and blood pressure values remained within the appropriate range for their ages. 

In our study, IVC-max and IVC-min significantly correlated with age, body weight, height, BSA, and BMI. It was also previously reported that there is a positive correlation between age and anthropometric measurements (weight, height, BMI, BSA, age), and sonographic IVC measurements [16,17]. However, IVC-DI and IVC-DV were also weakly and negatively correlated with anthropometric measurements.

There were several limitations to this study. The first limitation: it includes a specific population that does not have different racial and ethnic characteristics. The second limitation: a single center study design. The third limitation: if IVC/aorta ratio measurement could have been done to our participants, which would have contributed to the literature in terms of normative values. Because, in a previous study, 0.8 value was reported as normal for normative values for IVC-DI/aorta in euvolemic children under 3 years of age [24]. Originally, in the planning phase of the study, we did plan to determine the IVC-DI normative values. The fourth limitation: we could have evaluated the correlation of patients’ central venous pressure values with IVC-DI and IVC-DV. However, since these patients were undergoing ambulatory surgery, central venous catheter placement would have been an invasive procedure. 

## 5. Conclusion

According to our knowledge, our study is the largest series of children in the literature where IVC-DI measurements were made. Our IVC-DI mean values were measured lower than two previous children studies, which can be explained by the inclusion of younger children [10,14]. In ventilated patients, the IVC collapses in the thorax, but it distends out of the thorax (abdomen), and the IVC diameter increases in the abdominal region as the tidal volume increases. We performed IVC-DI and IVC-DV measurements in pediatric patients experiencing 5 cm H2O PEEP pressure and 7–8 mL/kg tidal volume. In future studies, these measurements can be performed at higher pressures and tidal volumes. In addition, more studies can be conducted in children where the response to fluid is evaluated.
